# Prenatal 1‐Nitropyrene Exposure Causes Autism‐Like Behavior Partially by Altering DNA Hydroxymethylation in Developing Brain

**DOI:** 10.1002/advs.202306294

**Published:** 2024-05-16

**Authors:** Ting Zhao, Cheng‐Qing Huang, Yi‐Hao Zhang, Yan‐Yan Zhu, Xiao‐Xi Chen, Tao Wang, Jing Shao, Xiu‐Hong Meng, Yichao Huang, Hua Wang, Hui‐Li Wang, Bo Wang, De‐Xiang Xu

**Affiliations:** ^1^ Department of Toxicology School of Public Health Anhui Medical University Hefei 230022 China; ^2^ Key Laboratory of Environmental Toxicology of Anhui Higher Education Institutes Anhui Medical University Hefei 230032 China; ^3^ School of Food and Bioengineering Hefei University of Technology Hefei 230009 China

**Keywords:** 1‐nitropyrene, autism‐like behavior, epigenetic reprogramming, hydroxymethylation, interneuron migration

## Abstract

Autism spectrum disorder (ASD) is a neurodevelopmental disorder, characterized by social communication disability and stereotypic behavior. This study aims to investigate the impact of prenatal exposure to 1‐nitropyrene (1‐NP), a key component of motor vehicle exhaust, on autism‐like behaviors in a mouse model. Three‐chamber test finds that prenatal 1‐NP exposure causes autism‐like behaviors during the weaning period. Patch clamp shows that inhibitory synaptic transmission is reduced in medial prefrontal cortex of 1‐NP‐exposed weaning pups. Immunofluorescence finds that prenatal 1‐NP exposure reduces the number of prefrontal glutamate decarboxylase 67 (GAD67) positive interneurons in fetuses and weaning pups. Moreover, prenatal 1‐NP exposure retards tangential migration of GAD67‐positive interneurons and downregulates interneuron migration‐related genes, such as *Nrg1*, *Erbb4*, and *Sema3F*, in fetal forebrain. Mechanistically, prenatal 1‐NP exposure reduces hydroxymethylation of interneuron migration‐related genes through inhibiting ten‐eleven translocation (TET) activity in fetal forebrain. Supplement with alpha‐ketoglutarate (α‐KG), a cofactor of TET enzyme, reverses 1‐NP‐induced hypohydroxymethylation at specific sites of interneuron migration‐related genes. Moreover, α‐KG supplement alleviates 1‐NP‐induced migration retardation of interneurons in fetal forebrain. Finally, maternal α‐KG supplement improves 1‐NP‐induced autism‐like behaviors in weaning offspring. In conclusion, prenatal 1‐NP exposure causes autism‐like behavior partially by altering DNA hydroxymethylation of interneuron migration‐related genes in developing brain.

## Graphical abstract

This study indicates that prenatal 1‐NP (1‐nitropyrene) exposure induces autism‐like behavior by retarding tangential migration of interneurons in fetal forebrain. Prenatal 1‐NP exposure downregulates interneuron migration‐related genes by altering DNA hydroxymethylation in developing brain. Prenatal 1‐NP exposure disrupts mitochondrial tricarboxylic acid cycle (TCA) and alpha‐ketoglutarate (α‐KG) synthesis. Maternal supplementation with α‐KG improves interneuron migration and autism‐like behavior in 1‐NP‐exposed offspring.

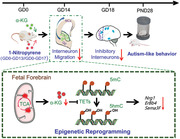

## 1 Introduction

Autism spectrum disorder (ASD) is a neurodevelopmental disorder, characterized by social communication disability and stereotypic behavior.^[^ ^1^ ^]^ The prevalence of ASD has risen from 0.76% to 1‐2.5% in the developed countries.^[^ ^2^, ^3^ ^]^ In China, the rate of ASD is approximately 0.7%–1%.^[^ ^4^, ^5^ ^]^ A multitude of studies have demonstrated that gestational exposure to environmental toxicants contributes to the rapidly increasing ASD rate in children.^[^ ^6^, ^7^, ^8^ ^]^ Several cohort studies confirmed that maternal exposure to motor vehicle exhausts increased the risk of ASD in children.^[^ ^9^, ^10^, ^11^ ^]^ 1‐Nitropyrene (1‐NP) is a characteristic nitro‐polycyclic aromatic hydrocarbon (nitro‐PAH) in diesel exhaust particles (DEPs) and cooking emissions.^[^ ^12^, ^13^ ^]^ An earlier report showed that gestational 1‐NP (10 µg kg^−1^) exposure during late pregnancy was associated with diminished learning and memory abilities in adolescent offspring.^[^ ^12^ ^]^ Moreover, gestational 1‐NP (10, 100 µg kg^−1^) exposure during the whole pregnancy caused anxiety‐like behavior in adult stage.^[^ ^14^ ^]^ Albeit these neurological disorders in association with 1‐NP exposure, it is still unclear whether maternal 1‐NP exposure induces autism‐like behavior in the offspring.

Increasing evidences indicated that interneurons in medial prefrontal cortex (mPFC) play pivotal roles in the modulation of social communication.^[^ ^15^ ^]^ Two reports showed that interneurons in the mPFC were decreased in ASD patients.^[^ ^16^, ^17^ ^]^ In rodents, interneurons originate in ganglionic eminence (GE) and tangentially migrate to neocortex in middle and late stages of brain development.^[^ ^18^, ^19^ ^]^ Recently, two studies indicated that interneuron migration retardation caused autism‐like behavior in mice.^[^ ^20^, ^21^ ^]^ It is generally accepted that several interneuron migration‐related molecules, including neuregulin1 (Nrg1) and semaphorin 3F (Sema3F), participate in regulating interneuron migration.^[^ ^22^ ^]^ Several studies demonstrate that hydroxymethylation modification is involved in the migration of placental trophoblast cells and neuronal stem cells.^[^ ^23^, ^24^ ^]^ Our previous study showed that Nrg1 could be transcriptionally regulated by modification of hydroxymethylation.^[^ ^14^ ^]^ Thus, we hypothesized that prenatal 1‐NP exposure restrains interneuron migration through inhibiting hydroxymethylation modification of interneuron migration‐associated genes.

Thus, this study was to investigate the impact of early‐life 1‐NP exposure on autism‐like behavior in a mouse model, and to unveil the potential role of interneuron migration in fetal forebrain. This study indicates that maternal 1‐NP exposure causes autism‐like behavior via retarding interneuron migration in fetal forebrain. Our research provides evidence that early‐life 1‐NP exposure retards interneuron migration partially by disrupting epigenetic reprogramming of interneuron migration‐associated genes in the developing brain.

## 2 Experimental Section

### 2.1 Materials

Anti‐GAD67 (198013) was bought from synaptic systems (Germany). Anti‐NeuN (ab177487), anti‐TET1 (ab191698), anti‐TET2 (ab213369) and anti‐TET3 (ab139311), anti‐SDHB (ab175225), and anti‐vATP5A (ab110413) were form Abcam (USA). 1‐NP (N22959) and alpha‐ketoglutarate (α‐KG, No.K1128) were supplied by Sigma Aldrich (Germany). Nuclear extraction reagents (78833) were from Thermo Fisher Scientific (USA). Ten eleven translocation (TET) enzymatic activity kit (P‐3086‐96) was from Epigentek (USA). All other regents were from Sigma Aldrich (Germany).

### 2.2 Animal Experiments

**Figure 1:**
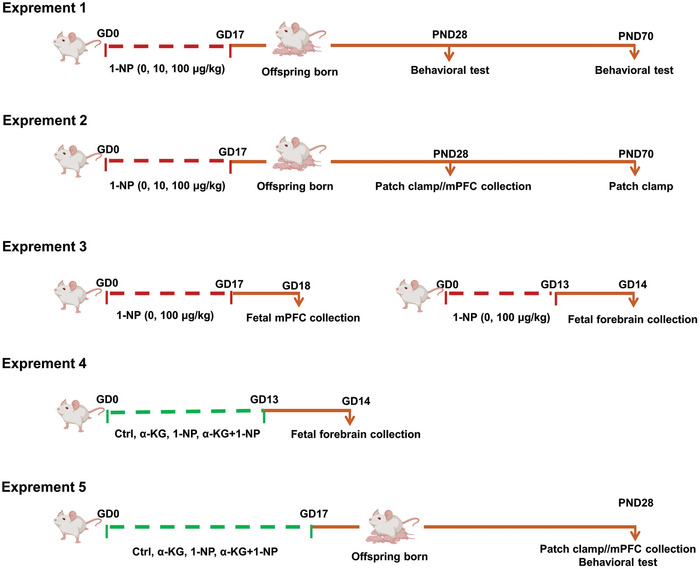
Experimental scheme for animals and treatments. Experiment 1, 30 pregnant mice were randomly assigned to three different groups. The pregnant mice orally received different dose of 1‐NP (0, 10, 100 µg kg^−1^) daily throughout pregnancy. All pregnant mice gave birth naturally. At PND28 and PND70, autism‐like behaviors were evaluated in offspring. Experiment 2, 10 pregnant mice were randomly assigned to two groups. The pregnant mice orally received two dose of 1‐NP (0, 100 µg kg^−1^) daily throughout pregnancy. All dams gave birth naturally. At PND28 or PND70, the offspring were sacrificed and the mPFC were collected for further experiments. Experiment 3, 10 pregnant mice were randomly assigned to two groups. The pregnant mice orally received two dose of 1‐NP (0, 100 µg kg^−1^) daily from GD0 to GD17. All dams were sacrificed on GD18. Fetal mPFC was collected for further experiments. On the other hand, another 10 pregnant mice were orally exposed to 1‐NP (100 µg kg^−1^) daily from GD0 to GD13. All dams were sacrificed on GD14. Fetal forebrain was collected for further experiments. Experiment 4, 20 pregnant mice were categorized into four groups: control (Ctrl), α‐KG, 1‐NP, and α‐KG+1‐NP. The pregnant mice were orally exposed to 1‐NP (100 µg kg^−1^) or α‐KG (2 g kg^−1^) daily from GD0 to GD13. All dams were sacrificed on GD14. Fetal forebrain was collected for further experiments. Experiment 5, the grouping of the 40 pregnant was the same as in Experiment 4 and the exposure time and method in pregnant mice were the same as Experiment 1. All dams were delivered spontaneously. Some PND28 offspring were sacrificed and mPFC were collected for further experiments. Autism‐like behaviors was also evaluated in PND28 offspring.

All mice were confirmed pregnant as method described in our previous study.^[^ ^12^ ^]^ In this study, animal experiments were divided into five parts. Experiment 1, 30 pregnant mice were randomly assigned to three different groups. Pregnant mice orally received different dose of 1‐NP (0, 10, 100 µg kg^−1^) daily throughout pregnancy. The dosage of 1‐NP referred to the previous study.^[^ ^14^ ^]^ All pregnant mice gave birth naturally. At postnatal day (PND)28, three‐chamber test was used to evaluate autism‐like behaviors in weaning pups. Moreover, at PND70, three‐chamber test was used to evaluate autism‐like behaviors in adult offspring. Experiment 2, 10 pregnant mice were randomly assigned to two groups. In the 1‐NP group, pregnant mice were received 100 µg kg^−1^ 1‐NP. In the control group, pregnant mice were given corn oil. The exposure time and method in pregnant mice were the same as Experiment 1. All pregnant mice gave birth naturally. At PND28, some weaning pups were sacrificed. The mPFC was collected for patch clamp, immunohistochemistry (IF), and Western blotting. At PND70, adult offspring were sacrificed. The mPFC was collected for patch clamp. Experiment 3, 10 pregnant mice were randomly assigned to two groups. In the 1‐NP group, pregnant mice were orally exposed to 1‐NP (100 µg kg^−1^) daily from gestational day (GD) GD0 to GD17. Pregnant mice from control group were given corn oil. All dams were sacrificed on GD18. Fetal mPFC was collected for IF and Western blotting. GD14 was the critical time point for migration of interneurons from ganglion eminence to fetal forebrain.^[^ ^18^ ^]^ In this study, 10 pregnant mice were randomly separated into two groups. In the 1‐NP group, pregnant mice were orally exposed to 1‐NP (100 µg kg^−1^) daily from GD0 to GD13. In the control group, pregnant mice were given corn oil. All dams were sacrificed on GD14. Fetal forebrain was collected for Western blot, IF, transcriptomic analysis, reverse transcription‐polymerase chain reaction (RT‐PCR), DNA hydroxymethylation and TET activity. Experiment 4, 20 pregnant mice were categorized into four groups: control (Ctrl), α‐KG, 1‐NP, and α‐KG+1‐NP. In the 1‐NP and 1‐NP+α‐KG groups, pregnant mice received oral exposure to 1‐NP (100 µg kg^−1^) daily from GD0 to GD13. In the α‐KG and 1‐NP+α‐KG groups, pregnant mice were given α‐KG (2 g kg^−1^) by gavage daily from GD0 to GD13. The dosage of α‐KG was based on previous studies.^[^ ^25^ ^]^ All dams were sacrificed on GD14. Fetal forebrain was collected for DNA hydroxymethylation, RT‐PCR and IF. Experiment 5, 40 pregnant were divided into four groups as in Experiment 4. The exposure time and method were the same as Experiment 1. All dams were delivered spontaneously. Some offspring were sacrificed and mPFC was collected for patch clamp, IF, and Western blotting. Three‐chamber social test was used to evaluate autism‐like behaviors in offspring. The animal experiments complied with the rules established by Anhui Medical University Animal Care and Use Committee (Ethical number: LLSC20190357). The detailed experimental protocol is shown in **Figure** **1**.

### 2.3 Behavioral Assessment

A three‐chamber apparatus with equal chambers was used to evaluate autism‐like behavior. In social behavior test, chambers on both sides were placed with two empty cages (E). An unfamiliar mouse (strange 1, S1), matched in age and gender, was placed in one cage. The test mice were allowed to explore three chambers freely for 10 min. Sniffing time in S1 or E was recorded by behavior software (Smart 3.0, RWD, China). In social novelty behavior test, another novel unfamiliar mouse (stranger 2, S2) was positioned in the remaining E after social behavior test. And test mice were given freedom to explore three chambers for 10 min. Sniffing time in S1 or S2 was recorded by behavior software.

### 2.4 Immunofluorescence (IF)

For weaning pups, mice were anesthetized and perfused with 4% polyformaldehyde. After embedded in optimal cutting temperature (OCT) compound, the brains were divided coronally into 30 µm thick sections. About 3 areas were randomly selected from each mPFC section for subsequent stages. After an hour blockage using goat serum at 37 °C, brain slices were subjected to incubate with GAD67 (1:1000) and NeuN (1:1000) for 24 h, respectively. Brain slices were counterstained with different secondary antibodies from different species. Nuclei were stained with Hoechst33342. The numbers of GAD67^+^ and NeuN^+^ neurons were analyzed by panoramic tissue cell quantitative analysis system (TissueFAXS Plus S, Austria). For fetal mice, brains were divided coronally into 40 µm thick sections. The slices of the ganglia bulge were identified. Three sections were stochastically selected from every ganglionic eminence and incubated with anti‐GAD67 (1:1000). After incubated with secondary antibody, nuclei were stained with Hoechst33342. The number of GAD67^+^ neurons was analyzed by panoramic tissue cell quantitative analysis system (TissueFAXS Plus S, Austria).

### 2.5 Mitochondrial Morphology Detection

Ganglionic eminence in fetal forebrain was selected and cut into 1 cubic millimeter tissue. The tissue was stabilized using 1% osmium tetroxide for 2 h at 4 °C. Following uranyl acetate staining, the tissue was dehydrated by gradient alcohol and acetone. Next, the tissue was infiltrated and embedded in embedding medium. The tissue was cut into 100 nm thick sections and stained with lead citrate. Transmission electron microscope was used to shoot mitochondria (Thermo Scientific Talos L120CG2, USA). The circumference of mitochondria and the percentage of abnormal mitochondria were counted in Image J (National Institutes of Health, USA).

### 2.6 Patch Clamp in the mPFC of Offspring

Fresh brain was prepared into 300 µm thick coronal slices in a 6% cold sucrose solution. The slice containing mPFC was transferred to artificial cerebrospinal fluid, filled with 95% O_2_ and 5% CO_2_, for 1 h. Tetrodotoxin (Sigma Aldrich) was applied to prevent action potential. 6‐cyano‐7‐nitroquinoxaline‐2,3‐dione (CNQX: No.C127, Sigma) was utilized to inhibit alpha‐amino‐3‐hydroxy‐5‐methyl‐4‐isoxazole‐propionic acid receptor (AMPA) receptor. Amino‐5‐ phosphonovaleric acid (APV: No.A8054, Sigma) was utilized to prevent N‐methyl‐D‐aspartate (NMDA) receptor. The miniature inhibitory postsynaptic current (mIPSC) of pyramidal neurons in the mPFC was detected in voltage clamp mode. The frequency and amplitude of mIPSC were analyzed by Mini 60 (Synaptosoft version 6.0.3, USA).

### 2.7 Measurement of 5hmC

APOBEC‐coupled epigenetic sequencing was used to detect 5hmC content in specific genes. In this method, 5hmC in specific genes was identified as “C,” while “C” and “5mC” were not. The level of 5hmC in specific genes was measured.^[^ ^26^ ^]^ The details are also described in Supporting Information.

### 2.8 TET Activity

Fresh fetal forebrain was used for nuclear protein extraction using specific reagent kits (78833, Thermo Scientific, USA). After adjusted to a suitable concentration, TET activity was detected by TET activity kit (P‐3086‐96, Epigentek, USA) according to manufacturer's instructions.

### 2.9 Determination of α‐KG

Fresh fetal forebrain underwent lysis in a mixture solution containing methanol, acetonitrile, and water (2:2:1). Following concentration adjustment, the supernatant with α‐KG was detected using liquid chromatography‐tandem mass spectrometry as formerly outlined.^[^ ^27^ ^]^

### 2.10 RNA Sequencing

Total RNAs were extracted from the matched sample using TRIzol reagent (Thermo Scientific). Total RNAs were used to establish RNA‐seq libraries using VAHTS mRNA‐seq V3 Library Prep Kit. Quality assessment was performed using BioAnalyzer and Qubit. All libraries were sequenced on the Illumina NovaSeq 6000 system. After quality control, sequence data were processed with spliced transcripts alignment to a reference (STAR) to produce read alignments. Raw read counts for annotated genes were subjected to normalization and analysis using DEseq2 v1.40.2. The resulting *P* < 0.05 and fold change of ≥1.5 was the threshold set for considering differential expression. The gene ontology (GO), Kyoto encyclopedia of genes and genomes (KEGG) and Reactome pathway enrichment analyses were performed using clusterProfiler package in R.

### 2.11 RNA Extraction and RT‐PCR

Total RNAs were extracted from fetal forebrain using reverse transcription system (Promega, USA). The extracted RNAs from fetal forebrain were converted into cDNA by reverse transcriptase. Quantitative RT‐PCR was implemented by LightCycler 480 SYBR Green I kit (Roche, USA). The list of primers targeting the specific gene is presented in Table S2 (Supporting Information).

### 2.12 Western Blot

Total proteins were obtained from either the mPFC or the forebrain. Dodecyl sulfate, sodium salt (SDS)–polyacrylamide gel electrophoresis (PAGE) was employed to separate protein. The protein was then transitioned to the polyvinylidene fluoride (PVDF) membrane. After incubating the primary and secondary antibodies, enhanced chemiluminescence (ECL) was used to test protein bands. The protein's optical density value was examined using Image J.

### 2.13 Statistical Analysis

Quantized data were displayed as means ± S.E.M. In two‐group experiments, student *t* test was used to detect statistical differences in the data with normal distribution and homogeneous variance. Adjusted‐*t* test was used to detect statistical differences in the data with normal distribution and uneven variance. In four‐group experiments, the differences were analyzed using two‐way analysis of variance (ANOVA) and Bonferroni test. 2^−ΔΔCt^ method was employed to analyze RT‐PCR results.

## 3 Results

### 3.1 Influence of Maternal 1‐NP Exposure on Autism‐Like Behavior in Offspring

**Figure 2:**
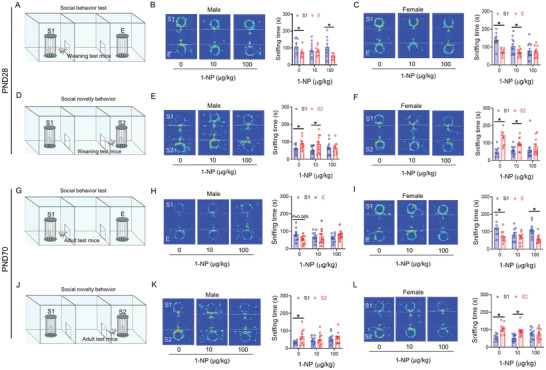
Influence of maternal 1‐NP exposure on autism‐like behavior in weaning offspring. 30 pregnant mice orally received different dose of 1‐NP (0, 10, 100 µg kg^−1^) daily from GD0 to GD17. All pregnant mice gave birth naturally. A–F) Three‐chamber social interaction test was used to evaluate autism‐like behaviors in weaning offspring (PND28). A) Pattern diagram of social behavior in weaning offspring. B) Sniffing time spent in male weaning offspring. C) Sniffing time spent in female weaning offspring. D) Pattern diagram of social novelty behavior in weaning offspring. E) Sniffing time spent in male weaning offspring. F) Sniffing time spent in female weaning offspring. G–L) Three‐chamber social interaction test was used to evaluate autism‐like behaviors in adult offspring (PND70). G) Pattern diagram of social behavior in adult offspring. H) Sniffing time spent in male adult offspring. I) Sniffing time spent in female adult offspring. J) Pattern diagram of social novelty behavior in adult offspring. K) Sniffing time spent in male adult offspring. L) Sniffing time spent in female adult offspring. *N* = 9–11. S1, stranger 1. S2, stranger 2. E, empty cage. *P < 0.05.

In social behavior test, test mice typically display a preference for interaction with stranger 1 mouse, spending longer time in sniffing stranger 1 mouse than in empty cage (**Figure** **2**A,G). In social novelty behavior test, test mice usually display a preference for interaction with stranger 2 mouse, spending longer sniffing time in stranger 2 mouse than in stranger 1 mouse (Figure 2D,J). In this study, social behavior test was performed in weaning offspring. Although weaning male mice from high‐dose group displayed a preference for interaction with stranger 1 mouse, this was not witnessed in the low‐dose group (Figure 2B). On the other hand, weaning female test mice from the high‐dose group did not display a preference for interaction with stranger 1 mouse (Figure 2C). Social novelty behavior test was then performed in weaning offspring. The result showed that weaning male test mice from low‐dose group displayed a preference for interaction with stranger 2 mouse, yet not for mice from the high‐dose group (Figure 2E). Weaning female test mice from high‐dose group displayed the similar phenotype as male mice received to high dose of 1‐NP (Figure 2F).

Next, social behavior and social novelty behavior were further evaluated in adult offspring. In social behavior test, adult male test mice from two 1‐NP groups did not display a preference for interaction with stranger 1 mouse (Figure 2H). On the other hand, adult female mice from the high‐dose group displayed a preference for interaction with stranger 1 mouse, but this was not observed in the low‐dose group (Figure 2I). In social novelty behavior, adult male test mice from two 1‐NP groups did not display a preference for interaction with stranger 2 mouse (Figure 2K). In addition, adult female mice from the low‐dose group displayed a preference for interaction with stranger 2 mouse, yet not for mice from the high‐dose group (Figure 2L).

### 3.2 Influence of Maternal 1‐NP Exposure on mIPSC in the mPFC of Offspring

**Figure 3:**
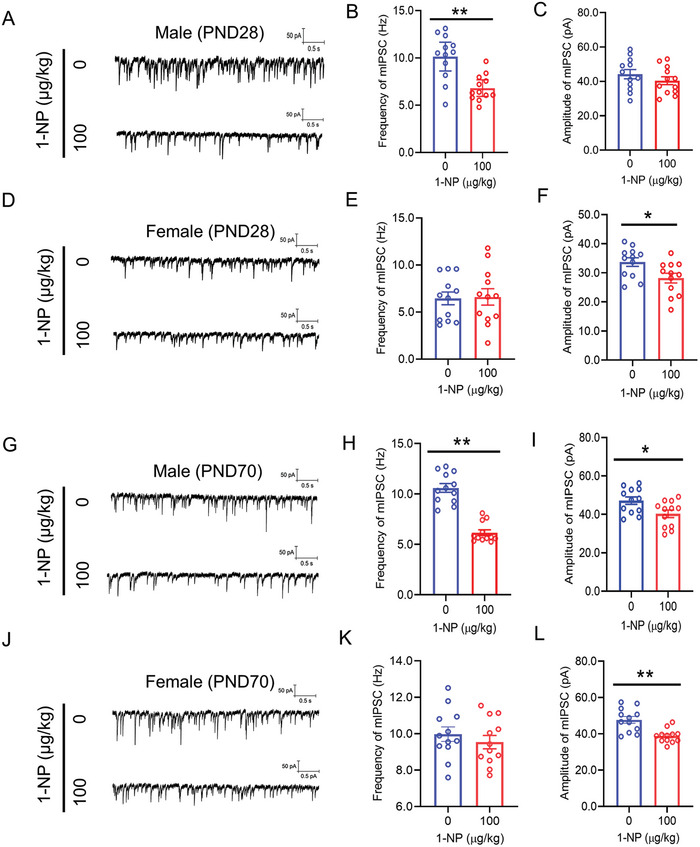
Influence of maternal 1‐NP exposure on mIPSC in offspring. 10 pregnant mice orally received different dose of 1‐NP (0, 100 µg kg^−1^) daily from GD0 to GD17. All pregnant mice gave birth naturally. A–F) Patch clamp was conducted on excitatory neurons to measure mIPSC in the mPFC of weaning offspring (PND28). A–C) mIPSC in the mPFC was measured in weaning male offspring. A) Representative photograph. B) Frequency of mIPSC. C) Amplitude of mIPSC. D–F) mIPSC in mPFC was measured in weaning female offspring. D) Representative photograph. E) Frequency of mIPSC. F) Amplitude of mIPSC. *N* = 12 excitatory neurons from 3 mice. *P < 0.05. **P < 0.01. G–L) Patch clamp was performed on excitatory neurons to measure mIPSC in the mPFC of adult offspring (PND70). G–I) mIPSC in the mPFC were measured in adult male offspring. G) Representative photograph. H) Frequency of mIPSC. I) Amplitude of mIPSC. J–L) mIPSC in the mPFC were measured in adult female offspring. J) Representative photograph. K) Frequency of mIPSC. L) Amplitude of mIPSC. *N* = 12 excitatory neurons from 3 mice. *P < 0.05. **P < 0.01.

Whole‐cell recording of excitatory neurons was used to evaluate mIPSC in the mPFC of weaning offspring. Although male offspring showed a diminished mIPSC frequency following 1‐NP exposure (Figure **3**A,B), no significant difference on the mIPSC amplitude was found between two groups (Figure 3A,C). Despite no difference on the mIPSC frequency (Figure 3D,E), gestational 1‐NP exposure caused diminished on the mIPSC amplitude in female weaning offspring (Figure 3D,F). Next, mIPSC was measured in the mPFC of adult offspring. The frequency and amplitude of mIPSC were both diminished in 1‐NP‐exposed male adult offspring (Figure 3G–I). Despite no change on the mIPSC frequency (Figure 3J,K), mIPSC amplitude in the mPFC was continued to be decreased in female adult offspring exposed 1‐NP (Figure 3J,L).

### 3.3 Influence of Maternal 1‐NP Exposure on GAD67^+^ Interneurons in the mPFC of Weaning Offspring

**Figure 4:**
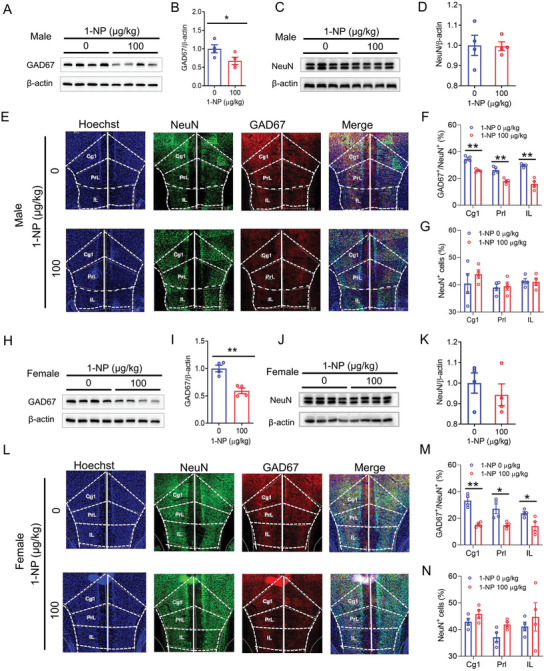
Influence of maternal 1‐NP exposure on GAD67^+^ interneurons in weaning offspring. 10 pregnant mice orally received different dose of 1‐NP (0, 100 µg kg^−1^) daily from GD0 to GD17. All pregnant mice gave birth naturally. A–G) Male offspring were euthanized on PND28 and their mPFC was harvested. A,B) GAD67 was performed by Western blot. C,D) NeuN was examined through Western blot. E) GAD67^+^ and NeuN^+^ neurons in each subfield of the mPFC were analyzed using IF. F) The proportion of GAD67^+^ to NeuN^+^ neurons in each subfield of the mPFC. G) The percentage of NeuN^+^ neurons in each subfield of the mPFC. Original magnification: 400×. *N* = 4. *P < 0.05. **P < 0.01. H–N) Female offspring was sacrificed on PND28 and the mPFC was collected. H,I) GAD67 was measured by Western blotting. J,K) NeuN was analyzed using Western blotting. L) GAD67^+^ and NeuN^+^ neurons in each subfield of the mPFC were analyzed using IF. M) The proportion of GAD67^+^ to NeuN^+^ neurons in each subfield of the mPFC. N) The percentage of NeuN^+^ neurons in each subfield of the mPFC. Original magnification: 400×. *N* = 4. *P < 0.05. **P < 0.01. Cg1, cingulate cortex, area 1. PrL, prelimbic cortex. IL, infralimbic cortex.

To investigate the influence of gestational 1‐NP exposure on GAD67^+^ interneurons, GAD67 in the mPFC, an interneuron mark,^[^ ^28^ ^]^ was detected in weaning male offspring. As expected, GAD67 protein in the mPFC was diminished in weaning male offspring exposed to 1‐NP (Figure **4**A,B). NeuN, a specific marker for neurons, was then detected in 1‐NP‐exposed weaning male offspring.^[^ ^29^ ^]^ The result showed that there was no difference on NeuN protein in the mPFC of two group offspring (Figure 4C,D). IF showed that the percentage of GAD67^+^ cells among NeuN^+^ neurons in each mPFC subregion (Cg1: cingulate cortex, area 1; PrL: prelimbic cortex; IL: infralimbic cortex) was diminished in weaning male offspring (Figure 4E,F). No difference on the percentage of NeuN^+^ neurons in each mPFC subregion was found between male offspring exposed to 1‐NP and controls (Figure 4E,G). The impact of maternal 1‐NP exposure on GAD67^+^ interneurons in the mPFC was detected in weaning female offspring. In female pups, the levels of GAD67 protein in the mPFC also showed reduced in pups exposed to 1‐NP (Figure 4H,I). No significant difference on NeuN protein in the mPFC was observed between 1‐NP and control female offspring (Figure 4J,K). As shown in Figure 4L,M, the proportion of GAD67^+^ to NeuN^+^ neurons in each mPFC subfield (Cg1, PrL, IL) was diminished. Although the percentage of NeuN^+^ neurons in PrL of mPFC was increased in pups exposed to 1‐NP, no significant difference in the percentage of NeuN^+^ neurons in Cg1 and IL of mPFC subregion was observed between 1‐NP and control female offspring (Figure 4L,N).

### 3.4 Influence of Gestational 1‐NP Exposure on Migration of Interneurons in Fetal Forebrain

**Figure 5:**
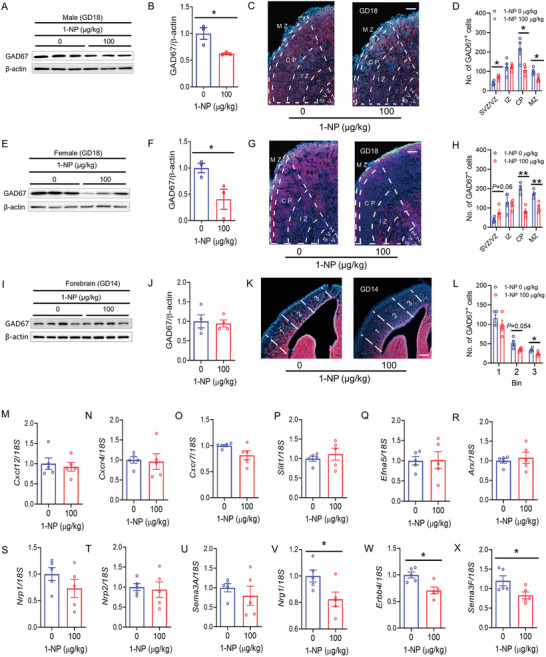
Influence of gestational 1‐NP exposure on migration of interneurons in fetal brain. A–H) 10 pregnant mice orally received different dose of 1‐NP (0, 100 µg kg^−1^) daily from GD0‐GD17. Fetal mPFC was collected on GD18. A,B) GAD67 protein in male fetuses was detected by Western blotting. C) GAD67^+^ interneurons in male fetuses were determined using IF. D) The distribution of GAD67^+^ interneurons was analyzed in different zones (MZ, CP, IZ, SVZ/VZ) of the mPFC. *N* = 4. Original magnification: 400×. *P < 0.05. E,F) The expression of GAD67 in female fetuses was measured using Western blotting. G) GAD67^+^ interneurons in female fetuses were determined using IF. H) The distribution of GAD67^+^ interneurons was analyzed in different zones (MZ, CP, IZ, SVZ/VZ) of mPFC. *N* = 4. Original magnification: 400×. *P < 0.05. **P < 0.01. I–X) 10 pregnant mice orally received different dose of 1‐NP (0, 100 µg kg^−1^) daily from GD0‐GD13. Fetal forebrains were collected on GD14. I,J) GAD67 in fetal forebrain was measured by Western blotting. K) GAD67 interneurons in forebrain cortex were determined using IF. L) GAD67 interneurons were analyzed in 1–3 areas of forebrain cortex. *N* = 4. Original magnification: 400×. *P < 0.05. M–X) Interneuron migration‐related genes in fetal forebrain were measured using RT‐PCR. M) *Cxcl12*. N) *Cxcr4*. O) *Cxcr7*. P) *Slit1*. Q) *Efna5*. R) *Arx*. S) *Nrp1*. T) *Nrp2*. U) *Sema3A*. V) *Nrg1*. W) *Erbb4*. X) *Sema3F*. *N* = 5. *P < 0.05. MZ, marginal zone; CP, cortical plate; IZ, intermediate zone; SVZ, subventricular zone; VZ, ventricular zone.

On GD18, GAD67 was detected in the mPFC of male fetus. As depicted in Figure **5**A,B, GAD67 protein in the mPFC was downregulated in 1‐NP‐exposed male fetus. Accordingly, GAD67^+^ interneurons in different layers (SVZ: subventricular zone; VZ: ventricular zone. IZ: intermediate zone; CP: cortical plate; MZ: marginal zone) was determined by IF. Although GAD67^+^ interneurons in the IZ layer showed no different between two groups, GAD67^+^ interneurons in the CP and MZ layers was diminished in the mPFC of in male fetus from the 1‐NP group (Figure 5C,D). By contrast, GAD67^+^ interneurons in the SVZ/VZ layers were increased in male fetus from the 1‐NP group (Figure 5C,D). Next, GAD67 was detected in the mPFC of female fetus. GAD67 protein in the mPFC was downregulated in 1‐NP‐exposed female fetus (Figure 5E,F). Although GAD67^+^ interneurons in the IZ layer showed no different between two groups, GAD67^+^ interneurons in the CP and MZ layers was diminished in female fetus from the 1‐NP group (Figure 5G,H). By contrast, GAD67^+^ interneurons in the SVZ/VZ layers were increased in female fetus from the 1‐NP group (Figure 5G,H). Previous studies indicate that mouse interneurons reach the frontal cortex through a tangential migration beginning at GD14.^[^ ^18^, ^30^ ^]^ In the current study, GAD67 in fetal forebrain was detected on GD14. Although prosencephalic GAD67 protein showed no different between two groups (Figure 5I,J), GAD67^+^ interneurons were reduced in middle and distal end (2 and 3 zones) of forebrain cortex in fetal forebrain from 1‐NP exposure group (Figure 5K,L).

Next, 12 interneuron migration genes were detected between two groups. Despite no difference on *Cxcl12*, *Cxcr4*, *Cxcr7, Slit1*, *Efna5, Arx, Nrp1*, *Nrp2* and *Sema3A* (Figure 5M–U), *Nrg1*, *Erbb4* and *Sema3F* showed a decrease in 1‐NP‐exposed fetal forebrain (Figure 5V–X). Interneuron differentiation‐related genes and proliferating cell nuclear antigen (PCNA) were then detected. As shown in Figure S1 (Supporting Information), prosencephalic PCNA protein showed no different between the 1‐NP and control groups. In addition, no difference on *Dlx1*, *Dlx2*, *Dlx5*, *NKX2‐1*, *NKX6‐2* and *LHX6*, six genes related to interneuron differentiation, was found between 1‐NP‐exposed fetal forebrain and control (Figure S2, Supporting Information).

### 3.5 Influence of Gestational 1‐NP Exposure on Hydroxymethylation of Interneuron Migration‐Related Genes in Fetal Forebrain

**Figure 6:**
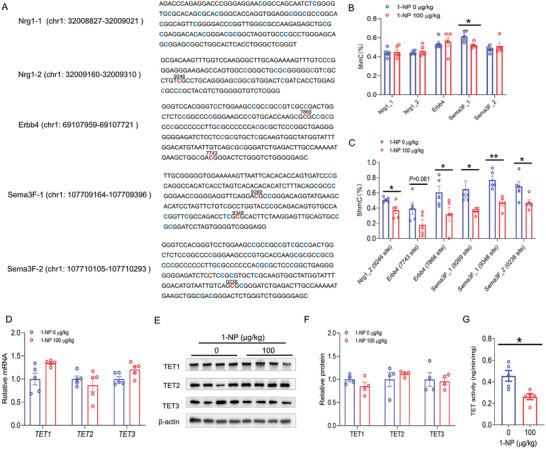
Influence of gestational 1‐NP exposure on hydroxymethylation of interneuron migration‐related genes in fetal forebrain. 10 pregnant mice orally received different dose of 1‐NP (0, 100 µg kg^−1^) daily from GD0‐GD13. Fetal forebrains were collected on GD14. A–C) The content of 5hmC at 5hmC‐rich CpG sites of interneuron migration‐related genes was measured in fetal forebrain. A) The sequences of 5hmC‐abundant CpG fragments of *Nrg1*, *Erbb4* and *Sema3F* genes. B) The content of 5hmC at 5hmC‐abundant CpG fragments of *Nrg1*, *Erbb4* and *Sema3F* genes. C) The content of 5hmC at 5hmC‐abundant CpG sites in *Nrg1*, *Erbb4* and *Sema3F* genes. *N* = 5. *P < 0.05. **P < 0.01. D) *TET1*, *TET2*, and *TET3* mRNAs in fetal forebrain were measured by RT‐PCR. *N* = 5. E,F) TET1, TET2, and TET3 proteins in fetal forebrain were analyzed by Western blotting. *N* = 4. G) TET activity in fetal forebrain was detected. *N* = 5. *P < 0.05.

The influence of gestational 1‐NP exposure on 5hmC content in interneuron migration‐related genes was measured in GD14 fetal forebrain. The sequences of methylation sites in *Nrg1*, *Erbb4* and *Sema3F* genes are presented in Figure **6**A. Although 5hmC level in CpG‐rich fragment of *Nrg1* and *Erbb4* genes showed no difference in two groups, prosencephalic 5hmC level in one CpG‐rich fragment of *Sema3F* gene was diminished in 1‐NP‐exposed fetuses (Figure 6B). The content of 5hmC in CpG sites of Nrg1, Erbb4 and Sema3F genes was then detected. As shown in Figure 6C, 5hmC content at CpG site (located on chr1:32009246) of *Nrg1* gene was diminished in fetal forebrain from the 1‐NP group. The content of 5hmC at two CpG sites (located on chr8: 69107743 and 69107866) of *Erbb4* gene was diminished in fetal forebrain from the 1‐NP group. And prosencephalic 5hmC level at three CpG sites (located on chr9: 107709269, 107709348 and 107710238) in *Sema3F* gene was diminished in fetus from the 1‐NP group. TET1, TET2 and TET3, three DNA demethylases, were measured in fetal forebrain. Although 1‐NP did not influence TET1, TET2, and TET3 expression (Figure 6D–F), prosencephalic TETs activity was diminished in fetus from the 1‐NP group (Figure 6G).

### 3.6 Influence of Gestational 1‐NP Exposure on Mitochondrial Function in Fetal Forebrain

**Figure 7:**
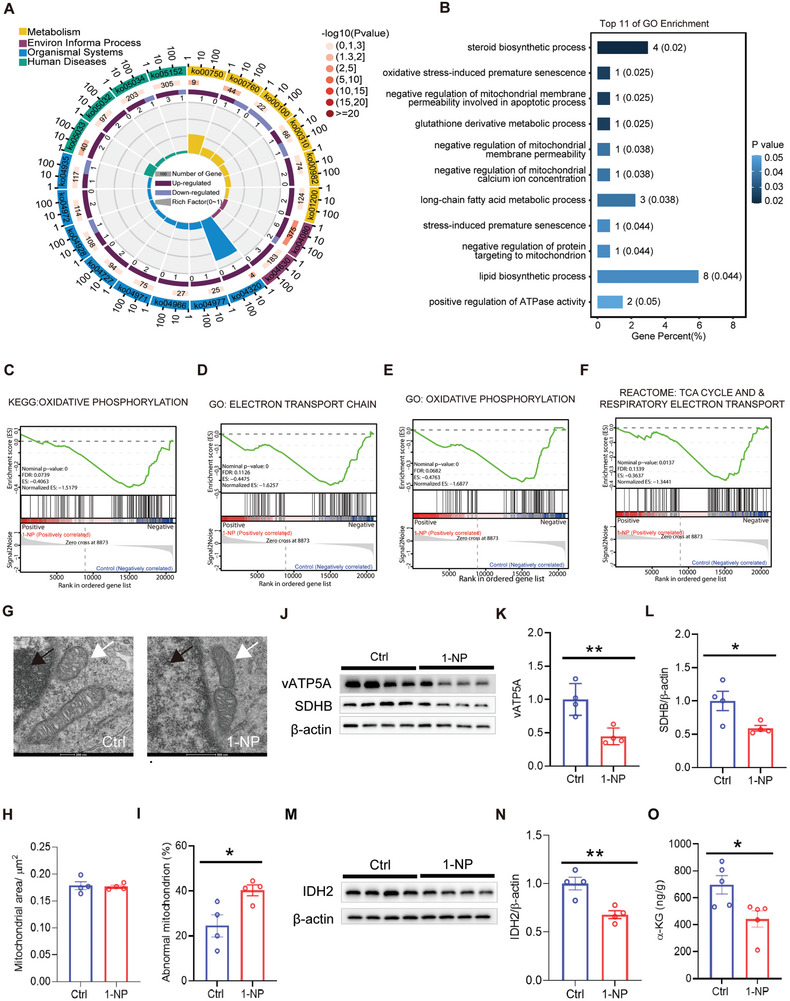
Influence of gestational 1‐NP exposure on mitochondrial function in fetal forebrain. 10 pregnant mice orally received different dose of 1‐NP (0, 100 µg kg^−1^) daily from GD0‐GD13. Fetal forebrains were collected on GD14. A–F) Transcriptomics of fetal forebrain was analyzed. A) KEGG pathway was used to analyze the differentially expressed genes. B) GO analysis of DEGs in mitochondrial metabolic‐related signal pathways. C–F) Mitochondrial function‐related signals were analyzed by GSEA. C) KEGG‐based GSEA: oxidative phosphorylation pathways. D,E) GO‐based GSEA: electronic transport chain and oxidative phosphorylation pathways. F) Reactome‐based GSEA: TCA cycle and respiratory electron transport. *N* = 5. *P* < 0.05. G–I) Fetal ganglionic eminence was separated from fetal forebrain. Mitochondrial microstructure in ganglionic eminence was evaluated. G) Representative picture of mitochondria. Original magnification: 13500×. H) Mitochondrial area. I) The percentage of abnormal mitochondria. *N* = 4. *P < 0.05. J–N) Mitochondrial related proteins in fetal forebrain were detected for Western blotting. J–L) vATP5A and SDHB, two oxidative phosphorylation‐related proteins, were measured. M,N) IDH2 was detected. *N* = 4. *P < 0.05. **P < 0.01. O) The level of α‐KG was determined using LC‐MS/MS. *N* = 5. *P < 0.05.

In order to explore the reason why 1‐NP‐induced hydroxymethylation of genes related to interneuron migration and inhibition of TET enzyme activity in fetal forebrain, transcriptome analysis was performed in GD14 fetal forebrain. KEGG and GO analyses found that 1‐NP caused prosencephalic mitochondrial related metabolic dysfunction, such as nicotinate/nicotinamide metabolism, steroid biosynthesis and the metabolism of long‐chain fatty acids (Figure **7**A,B). KEGG‐based gene set enrichment analysis (GSEA) showed that oxidative phosphorylation pathways were negatively enriched in 1‐NP‐exposed fetal forebrain (Figure 7C). GO‐based GSEA showed that electronic transport chain and oxidative phosphorylation pathways were negatively enriched in the forebrain of fetuses exposed to 1‐NP (Figure 7D,E). Reactome‐based GSEA showed that tricarboxylic acid (TCA) cycle and respiratory electron transport were negatively enriched in 1‐NP‐exposed fetal forebrain (Figure 7F). Moreover, despite no difference in mitochondrial area, the cristae structure of mitochondria in ganglionic eminence, the origin site of interneuron, was disappeared in 1‐NP‐exposed fetal forebrain (Figure 7G–I). Succinate dehydrogenase (SDH) B and vATP5A, two oxidative phosphorylation‐related proteins, were down‐regulated in 1‐NP‐induced fetal forebrain (Figure 7J–L). Finally, isocitrate dehydrogenase 2 (IDH2), the key enzyme for mitochondrial α‐KG synthesis, and α‐KG, a TET co‐factor, were reduced in fetal forebrain from the 1‐NP group (Figure 7M–O).

### 3.7 Effect of Supplementation with α‐KG on 1‐NP‐Evoked Hypohydroxymethylation of Interneuron Migration‐Related Genes in Fetal Forebrain

**Figure 8:**
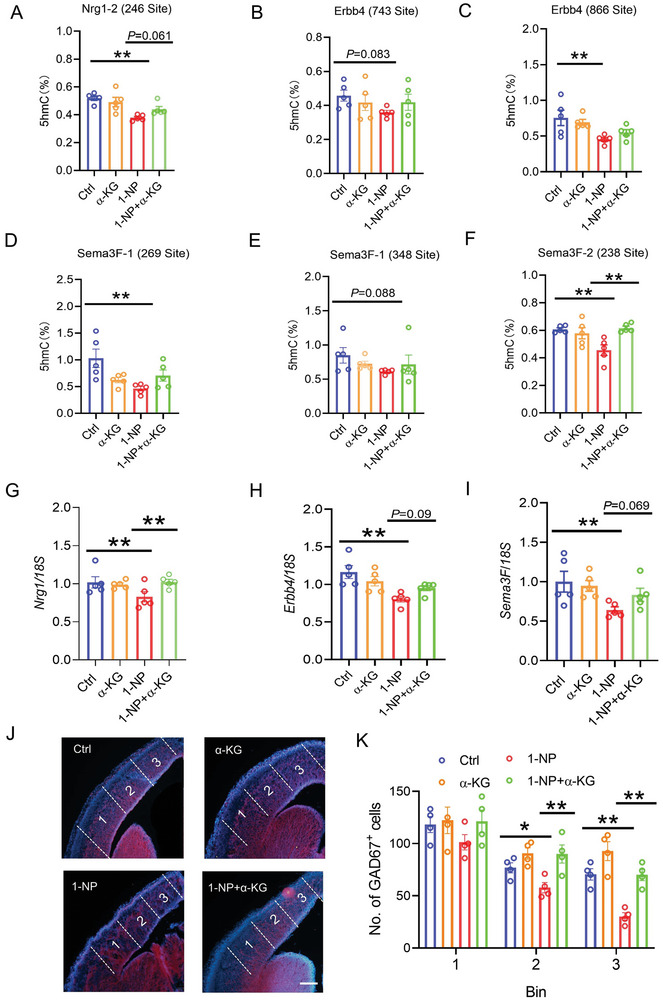
Effects of supplementation with α‐KG on 1‐NP‐evoked hypohydroxymethylation of interneuron migration‐related genes and interneuron migration inhibition in fetal forebrain. 20 pregnant mice orally received different dose of 1‐NP (0, 100 µg kg^−1^) daily from GD0‐GD13. Fetal forebrains were collected on GD14. A–F) The content of 5hmC at 5hmC‐rich CpG sites of interneuron migration‐related genes was measured in fetal forebrain. A) The content of 5hmC in the site (chr1: 32009246) of *Nrg1* gene. B,C) The content of 5hmC in two CpG sites (chr8: 69107743 and 69107866) of *Erbb4* gene. D‐F) The content of 5hmC in three CpG sites (chr9: 107709269, 107709348 and 107710238) of *Sema3F* gene. *N* = 5. **P < 0.01. G–I) The mRNAs of interneuron migration‐related genes in fetal forebrain were measured using RT‐PCR. G) *Nrg1*. H) *Erbb4*. I) *Sema3F. N* = 5. *P < 0.05. **P < 0.01. J,K) The number of GAD67 interneurons was measured in 1–3 areas of forebrain cortex. *N* = 4. **P < 0.01.

The effect of supplementation with α‐KG on 1‐NP‐evoked hypohydroxymethylation of interneuron migration‐related genes is presented in Figure **8**A–F. As expected, gestational supplementation with α‐KG inhibited 1‐NP‐evoked decrease in 5hmC at the CpG site (chr1: 32009246) in *Nrg1* gene (Figure 8A). Moreover, gestational supplementation with α‐KG prevented 1‐NP‐evoked decrease in 5hmC at one CpG sites (chr9:107710238) of *Sema3F* gene (Figure 8F). Supplementation with α‐KG had a protective trend against 1‐NP‐induced decrease in 5hmC at Erbb4 or other Sema3F CpG sites (Figure 8B–E). Next, the effects of α‐KG on 1‐NP‐caused decrease in the expression of interneuron migration‐related genes were analyzed. As shown in Figure 8G–I, gestational supplementation with α‐KG alleviated 1‐NP‐induced decrease in *Sema3F, Nrg1*, and *Erbb4* in fetal forebrain (Figure 8G–I).

### 3.8 Effects of Supplementation with α‐KG on 1‐NP‐Induced Interneuron Migration Inhibition

**Figure 9:**
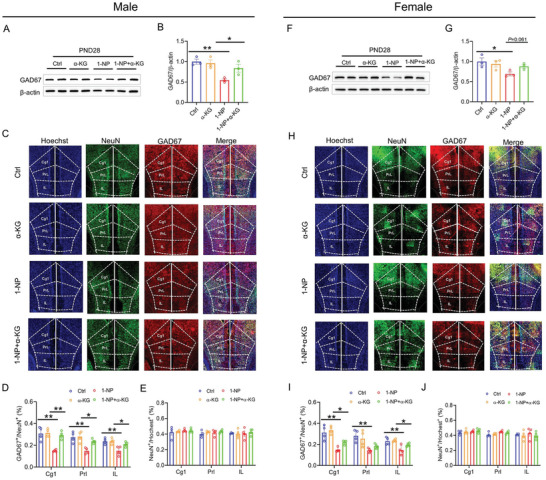
Effects of gestational α‐KG supplementation on interneurons in weaning offspring. 20 pregnant mice orally received different dose of 1‐NP (0, 100 µg kg^−1^) daily from GD0‐GD17. All pregnant mice gave birth naturally. A‐E) Weaning male offspring was sacrificed on PND28 and the mPFC was harvested. A,B) GAD67 was measured by Western blotting. C–E) GAD67^+^ and NeuN^+^ neurons in mPFC were measured by IF. C) Representative photograph. D) The proportion of GAD67^+^ to NeuN^+^ neurons in each subfield of the mPFC. E) Percentage of NeuN^+^ neurons in the mPFC. *N* = 4. Original magnification: 400×. *P < 0.05. **P < 0.01. F–J) Weaning female offspring was sacrificed on PND28 and the mPFC was collected. F,G) GAD67 was measured by Western blotting. H–J) GAD67^+^ and NeuN^+^ neurons in the mPFC were measured using IF. H) Representative photograph. I) The proportion of GAD67^+^ to NeuN^+^ neurons in each subfield of the mPFC. J) Percentage of NeuN^+^ neurons in the mPFC. *N* = 4. Original magnification: 400×. *P < 0.05. **P < 0.01.

The effects of α‐KG supplements on 1‐NP‐caused interneuron migration inhibition were analyzed. As shown in Figure 8J,K, gestational supplementation with α‐KG reversed 1‐NP‐caused decrease in GAD67^+^ interneurons in the middle and distal end of forebrain cortex. The effects of gestational α‐KG supplementation on GAD67^+^ interneurons in the mPFC were then evaluated in weaning male offspring. As depicted in Figure **9**A,B, gestational supplementation with α‐KG prevented 1‐NP‐evoked downregulation of GAD67 protein in the mPFC. Despite minimal effect on NeuN^+^ neurons (Figure 9C,E), gestational supplementation with α‐KG reversed 1‐NP‐induced decrease in the proportion of GAD67^+^ to NeuN^+^ neurons in the mPFC (Figure 9C,D). Next, the effects of gestational α‐KG supplementation on GAD67^+^ interneurons in the mPFC were analyzed in weaning female offspring. As depicted in Figure 9F,G, 1‐NP‐induced reduction of GAD67 protein in the mPFC was reversed by α‐KG supplementation. Despite no significant difference on the percentage of NeuN^+^ neurons in each subfield of the mPFC (Figure 9H,J), 1‐NP‐induced decrease in the proportion of GAD67^+^ to NeuN^+^ neurons in Cg1 and IL subfield of the mPFC was reversed in α‐KG‐supplemented female offspring (Figure 9H,I).

### 3.9 Effects of Supplementation with α‐KG on 1‐NP‐Induced mIPSC Transmission Disorder in Offspring

**Figure 10:**
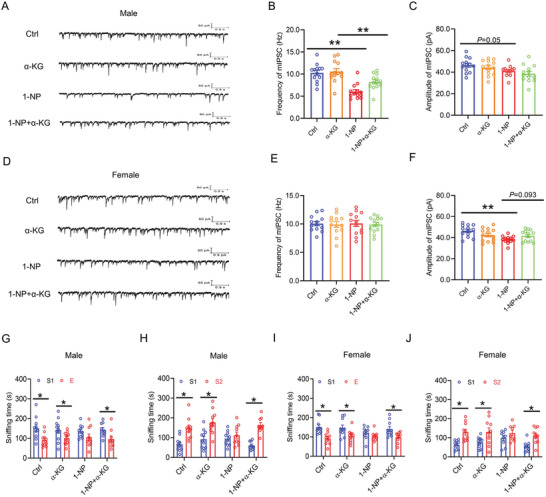
Effects of gestational α‐KG supplementation on 1‐NP‐induced mIPSC transmission disorder and autism‐like behaviors. 20 pregnant mice orally received different dose of 1‐NP (0, 100 µg kg^−1^) daily from GD0 to GD17. All pregnant mice gave birth naturally. A–C) mIPSC in the mPFC was measured in male offspring. A) Representative photograph. B) Frequency of mIPSC. C) Amplitude of mIPSC. *N* = 13 excitatory neurons from three mice. **P < 0.01. D–F) mIPSC in the mPFC was measured in female offspring. D) Representative photograph. E) Frequency of mIPSC. F) Amplitude of mIPSC. *N* = 13 excitatory neurons from three mice. **P < 0.01. G–J) Three‐chamber social test was used to evaluate autism‐like behavior in offspring. G) Sniffing time spent in sociability test in male offspring. H) Sniffing time spent in social novelty test in male offspring. I) Sniffing time spent in sociability test in female offspring. J) Sniffing time spent in social novelty test in female offspring. *N* = 9‐11. *P < 0.05.

The effects of maternal α‐KG supplementation on 1‐NP‐caused mIPSC transmission disorder in the mPFC were analyzed in male offspring. Despite no impact on mIPSC amplitude (Figure **10**A,C), gestational supplementation with α‐KG was able to rescue 1‐NP‐induced decrease in mIPSC frequency in male offspring (Figure 10A,B). The effects of gestational α‐KG supplementation on 1‐NP‐induced mIPSC transmission disorder in the mPFC were then analyzed in female offspring. The results showed no difference on mIPSC frequency among four female groups (Figure 10D,E). Interestingly, gestational supplementation with α‐KG was able to protect 1‐NP‐evoked decrease in mIPSC amplitude in the mPFC (Figure 10D,F).

### 3.10 Effects of Supplementation with α‐KG on 1‐NP‐Caused Autism‐Like Behaviors in Offspring

The effects of gestational α‐KG supplementation on 1‐NP‐induced autism‐like behavior were assessed in male offspring. The result showed that gestational supplementation with α‐KG prevented social behavior disorder in male offspring (Figure 10G). And gestational supplementation with α‐KG alleviated social novelty behavior disorder in male offspring (Figure 10H). Next, the effects of gestational α‐KG supplementation on 1‐NP‐evoked autism‐like behavior were assessed in female offspring. The result showed that gestational supplementation with α‐KG was able to protect social behavior disorder in female offspring (Figure 10I). Additionally, gestational supplementation with α‐KG was able to protect social novelty behavior disorder in female offspring (Figure 10J).

## 4 Discussion

The aim of our study was to evaluate the influence of prenatal 1‐NP exposure on autism‐like behaviors in a mouse model. The results showed that prenatal 1‐NP exposure caused autism‐like behavior in the offspring during weaning and adult period in a gender‐specific manner, and such neurobehavioral gender difference was more pronounce in the males than their female counterparts particularly at later adult stage. Patch clamp observed a dysfunction of mIPSC in the mPFC of 1‐NP‐exposed weaning and adult mice. Furthermore, interneurons in the mPFC were diminished in weaning offspring exposed to 1‐NP. Tangential migration of interneurons from ganglion eminence to forebrain cortex was deferred in fetuses from the 1‐NP group. These findings potentially indicate that early‐life 1‐NP exposure causes autism‐like behavior by retarding migration of interneurons in the developing brain.

Several cohort studies confirmed that gestational exposure to motor vehicle exhaust increased the risk of ASD in offspring.^[^ ^9^, ^10^ ^]^ 1‐NP, a characteristic nitro‐PAH, mainly derives from diesel engine exhaust particles.^[^ ^31^ ^]^ In this study, we used 1‐NP to construct an animal model of exposure to nitro‐PAH during pregnancy. Our results showed for the first time that prenatal exposure to 1‐NP induced autism‐like behavior in offspring. It is widely accepted the prevalence of autism in boys is higher than in girls.^[^ ^32^ ^]^ An earlier case‐control study indicated that the association of ASD with mercury was observed only in girls but not in boys.^[^ ^33^ ^]^ A cohort study showed that prenatal exposure to diesel engine exhaust particles was more strongly associated with autism in boys than in girls.^[^ ^34^ ^]^ In this study, in social behavior test, male and female weaning offspring exhibited social behavior disorders in different doses of 1‐NP exposure groups. Yet, there was no gender difference in social novelty behavior disorders induced by 1‐NP. ASD is generally considered a lifelong illness.^[^ ^35^ ^]^ The diagnosis of adult autism and the quality of life in adult autism patients were received an increasing attention.^[^ ^35^, ^36^ ^]^ To explore whether 1‐NP‐induced autism‐like behaviors could persist into adulthood, we detected autism‐like behaviors in adult offspring. We observed that social behavior disorder and social novelty behavior disorder were present in both doses of 1‐NP for the adult male mice, whereas female adult mice had much attenuated effect. These results provide experimental evidence that prenatal 1‐NP exposure causes autism‐like behavior in a gender‐specific manner.

The mPFC is the major brain region for regulating social communication.^[^ ^37^ ^]^ Increasing evidences indicate that disruption in the GABAergic signaling is involved in the pathogenesis of ASD.^[^ ^38^, ^39^ ^]^ Small inhibitory postsynaptic currents (mIPSCs) is one of GABAergic signals, spontaneously released by GABAergic neurons.^[^ ^40^ ^]^ According to a recent report, the frequency of GABAergic mIPSC was reduced in somatosensory cortex of valproic acid‐induced ASD.^[^ ^41^ ^]^ In our study, the frequency of GABAergic mIPSC were reduced in the mPFC of 1‐NP‐exposed male weaning pups. And the amplitude of GABAergic mIPSC were reduced in the mPFC of 1‐NP‐exposed female weaning pups. Interestingly, 1‐NP‐induced inhibitive synaptic transmission disorder could continue until adulthood. It is widely accepted that interneurons is the main GABAergic neurons in the mPFC.^[^ ^42^ ^]^ GAD67, a maker for interneurons, is the rate limiting enzyme for GABA synthesis.^[^ ^28^, ^43^ ^]^ The clinical data indicated that GAD67 was reduced in parietal cortex of ASD patients.^[^ ^44^ ^]^ Animal experiments showed that GAD67 deficiency impaired social behaviors in mice.^[^ ^20^, ^45^ ^]^ This study found that GAD67 protein in the mPFC was downregulated in 1‐NP‐exposed weaning offspring. GAD67^+^ interneurons were reduced in each mPFC subregion of 1‐NP‐exposed weaning offspring. Future work will be preformed to investigate the effect of 1‐NP on GAD67 in the mPFC of adult offspring.

Prefrontal interneurons are derived from progenitors located in the ganglionic eminences of fetal forebrain.^[^ ^46^ ^]^ From GD14 to GD18, interneurons migrated tangentially from ganglionic eminences to fetal forebrain cortex.^[^ ^18^, ^30^ ^]^ Several studies indicated that interneuron migration retardation could induce autism‐like behaviors.^[^ ^20^, ^21^ ^]^ In this current study, we further analyzed the influence of gestational 1‐NP exposure on GAD67^+^ interneurons in fetal mPFC and forebrain. On GD18, GAD67 protein and GAD67^+^ interneurons in the CP and MZ layers of mPFC were diminished in 1‐NP‐exposed fetus. On GD14, GAD67^+^ neurons in middle and distal ends of forebrain cortex were reduced in fetuses from the 1‐NP group. These findings potentially indicate that maternal 1‐NP exposure during pregnancy induces interneuron migration retardation in fetal forebrain.

Increasing data indicate that various interneuron migration‐related molecules are involved in the modulation of interneuron migration.^[^ ^47^ ^]^ Nrg1, a classic chemical attractant, and its receptor Erbb4, are necessary and sufficient to promote tangential migration of interneurons to the cortex.^[^ ^48^ ^]^ During interneuron migration, chemical repellants migrate around and avoid the striatum before entering neocortex. Sema 3A/3F, two representative chemical repellants, collaborate with their receptors Neuropilin 1/2 (Nrp1/2) to avoid interneuron migration to striatum.^[^ ^49^ ^]^ An epidemiological report showed that Nrg1 was decreased in the blood of ASD patients.^[^ ^50^ ^]^ Two animal experiments proved that Sema3F deletion reduced interneurons in fetal forebrain.^[^ ^51^, ^52^ ^]^ Other chemical attractants including Arx and Cxcl12 and two chemical repellants Slit1/Efna5 are also involved in tangential migration of interneurons.^[^ ^53^, ^54^, ^55^, ^56^ ^]^ In the current study, we observed a reduction of mRNA level in *Nrg1* and its receptor *Erbb4* in the fetal forebrain exposed to 1‐NP. Moreover, mRNA level of *Sema3F* was reduced, whereas no significant change was observed in Sema3A, Nrp1/2, Arx, Cxcl12 and its receptor Cxcr4, Slit1 and Efna5 mRNAs in 1‐NP‐exposed fetal forebrain. Several studies indicate that *Dlx1*, *Dlx2*, *Dlx5*, *Nkx2‐1*, *Nkx6‐2* and *LHX6*, six interneuron differentiation‐related genes, are associated with ASD.^[^ ^57^, ^58^, ^59^, ^60^, ^61^ ^]^ Moreover, excessive cortical proliferation is involved in the pathogenesis of ASD.^[^ ^62^, ^63^ ^]^ In this study, neither PCNA protein nor interneuron differentiation‐related gene was changed in 1‐NP‐exposed fetal forebrain. Altogether, these findings potentially indicate that 1‐NP‐caused a reduction of mRNA level in interneuron migration‐related genes may contribute to migration retardation of interneurons in fetal forebrain.

Several studies confirmed that hydroxymethylation, a new epigenetic modification, was involved in the modulation of cell migration‐related genes.^[^ ^64^, ^65^, ^66^ ^]^ An in vitro report showed that knockout of DNA hydroxymethylase reduced Nrg1 expression in prostate cancer cells.^[^ ^67^ ^]^ By contrast, overexpression of DNA hydroxymethylase elevated Erbb4 abundance in hepatocellular carcinoma cells.^[^ ^68^ ^]^ In this study, we found that 5hmC content at one CpG site (located on chr1: 32009246) in *Nrg1* gene was diminished in fetal forebrain from the 1‐NP group. Moreover, prosencephalic 5hmC content at two CpG sites (located on chr8: 69107866 and 69107899) in *Erbb4* gene was decreased in fetus from the 1‐NP group. In addition, prosencephalic 5hmC content at three CpG sites (located on chr9: 107709348, 107709269 and 107710238) in *Sema3F* gene was decreased in fetus from the 1‐NP group. Three TET enzymes. TET1, TET2 and TET3, are involved in regulation of DNA hydroxymethylation.^[^ ^69^ ^]^ An earlier study showed that TET1, TET2 and TET3 were highly expressed in fetal neocortex.^[^ ^70^ ^]^ Recently, an epidemiological study observed autism phenotypes in patients with human Mendelian disorder with TET3 deficiency.^[^ ^71^ ^]^ Moreover, TET1‐deficient mice exhibited autism‐like behaviors.^[^ ^72^ ^]^ In this study, we detected TETs expression and TETs activity in fetal forebrain. Although the expression of TETs remained unchanged, TET enzymatic activity was reduced in 1‐NP‐exposed fetal forebrain. These findings potentially indicate that gestational 1‐NP exposure causes hypohydroxymethylation of interneuron migration‐related genes via inhibiting TETs enzymatic activity in fetal brain. Several studies have demonstrated that α‐KG, an intermediate of TCA cycle, is a co‐factor for TET enzymes.^[^ ^73^, ^74^ ^]^ Isocitrate dehydrogenase 2 (IDH2) facilitates the transformation of isocitric acid to α‐KG within the TCA cycle.^[^ ^75^ ^]^ Recently, we found that 1‐NP suppressed mitochondrial respiratory function and oxidative phosphorylation in testicular Leydig cells.^[^ ^76^ ^]^ In this study, KEGG pathway showed that mitochondrial metabolisms, including nicotinate/nicotinamide metabolism, steroid biosynthesis and long‐chain fatty acid metabolism, were disturbed in 1‐NP‐exposed fetal forebrain. Moreover, electronic transport chain and oxidative phosphorylation pathways were markedly negatively enriched in fetal forebrain from the 1‐NP group. Electron microscopy results revealed that mitochondrial cristae structure in ganglionic eminence was disappeared in 1‐NP‐exposed fetal forebrain. Two oxidative phosphorylation‐related proteins, SDHB and vATP5A, were diminished in 1‐NP‐exposed fetal forebrain. IDH2 protein and its metabolite α‐KG were reduced in 1‐NP‐exposed fetal forebrain. To explore the role of mitochondrial dysfunction on TETs activity, pregnant mice were supplemented with α‐KG. Interestingly, gestational supplementation with α‐KG was able to rescue 1‐NP‐induced hypohydroxymethylation in the CpG sites of interneuron migration‐related genes. In addition, gestational supplementation with α‐KG prevented 1‐NP‐caused decrease in *Nrg1*, *Erbb4*, and *Sema3F* mRNAs in fetal forebrain. These results suggest that gestational 1‐NP exposure alters epigenetic reprogramming of interneuron migration‐related genes by disturbing mitochondrial TCA metabolism in the developing brain. Numerous clinical reports observed intestinal flora disturb in ASD patients.^[^ ^77^, ^78^, ^79^ ^]^ Indeed, an early study indicated that gut microbiota were involved in nitro‐PAH metabolism.^[^ ^80^ ^]^ Thus, next work is required to further explore the role of gut microbiota on 1‐NP‐evoked autism‐like behaviors.

These findings have significant clinical and preventive implications. Indeed, α‐KG metabolism is involved in epigenetic reprogramming during early embryo development.^[^ ^81^ ^]^ A recent study demonstrated that α‐KG metabolic disorders caused recurrent spontaneous miscarriage through impairing decidualization.^[^ ^82^ ^]^ By contrast, gestational α‐KG supplementation prevented spontaneous abortion through improving trophoblast invasion in mice.^[^ ^83^ ^]^ In this study, we found that gestational supplementation with α‐KG prevented 1‐NP‐caused migration retardation of interneurons in fetal forebrain. Moreover, gestational α‐KG supplementation prevented 1‐NP‐induced autism‐like behavior in offspring. These findings will establish a theoretical basis on clinical application of α‐KG for preventing environmental toxicant‐induced developmental diseases.

In summary, this study investigated the impact of prenatal exposure to 1‐NP, a representative nitro‐PAH, on autism‐like behavior in offspring. Our results indicate that prenatal 1‐NP exposure causes autism‐like behavior by retarding tangential migration of interneurons from ganglion eminence to forebrain cortex. Moreover, prenatal 1‐NP exposure downregulates interneuron migration‐related genes through altering epigenetic reprogramming in fetal forebrain. Mechanistically, prenatal 1‐NP exposure disrupts mitochondrial TCA and α‐KG synthesis. Supplementation with α‐KG, an intermediate of mitochondrial TCA cycle, improves hydroxymethylation reprogramming of interneuron migration‐related genes. Maternal supplementation with α‐KG improves interneuron migration and autism‐like behavior in 1‐NP‐exposed offspring.

## Conflict of Interest

The authors declare no conflict of interest.

## Supporting information

Supporting Information

Supporting Information

Supporting Information

## Data Availability

Research data are not shared.
