# Supplementary material for: Prenatal 1‐Nitropyrene Exposure Causes Autism‐Like Behavior Partially by Altering DNA Hydroxymethylation in Developing Brain
Source: Adv Sci (Weinh). 2024 May 16;11(28):2306294. doi: 10.1002/advs.202306294 (PMC11267330; doi:10.1002/advs.202306294)
Supplement: Supplementary file 1 — Supporting Information [file ADVS-11-2306294-s002.pdf]

ADVANCED SCIENCE
Open Access

Supporting Information

for *Adv. Sci.*, DOI 10.1002/advs.202306294

Prenatal 1-Nitropyrene Exposure Causes Autism-Like Behavior Partially by Altering DNA Hydroxymethylation in Developing Brain

Ting Zhao, Cheng-Qing Huang, Yi-Hao Zhang, Yan-Yan Zhu, Xiao-Xi Chen, Tao Wang, Jing Shao, Xiu-Hong Meng, Yichao Huang, Hua Wang, Hui-Li Wang, Bo Wang and De-Xiang Xu**

Supplementary data

Method

Measurement of 5hmC

APOBEC-coupled epigenetic sequencing (ACE-Seq) was used to detected 5hmC content in specific genes. Multiplex PCR of *Nrg1*, *Erbb4* and *Sema3F* genes was performed. The primers were listed in supplemental Table S1. PCR products were then performed with T4 Phage β-glucosyltransferase and APOBEC3A (A3A). Following deamination, the products were sequenced using the Accel Methyl-NGS kit (Swift Biosciences, Inc). Bismark (v0.14.3) was used to map the trimmed reads to the reference genomes. For each cytosine within CG dinucleotides, “C” bases from ACE-Seq were considered to be 5hmC. By contrast, “T” bases were considered to be methylated or unmodified cytosines.

Figures

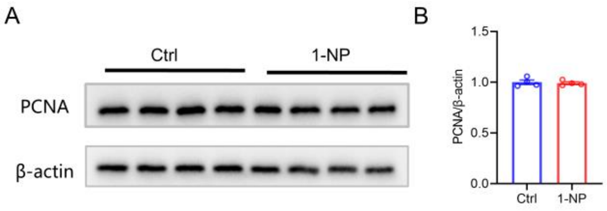

Fig.S1. Influence of gestational 1-NP exposure on PCNA protein in fetal forebrain. PCNA, a maker for cell proliferation, was determined by Western blotting. N=4. t=0.317, df=6, P=0.762.

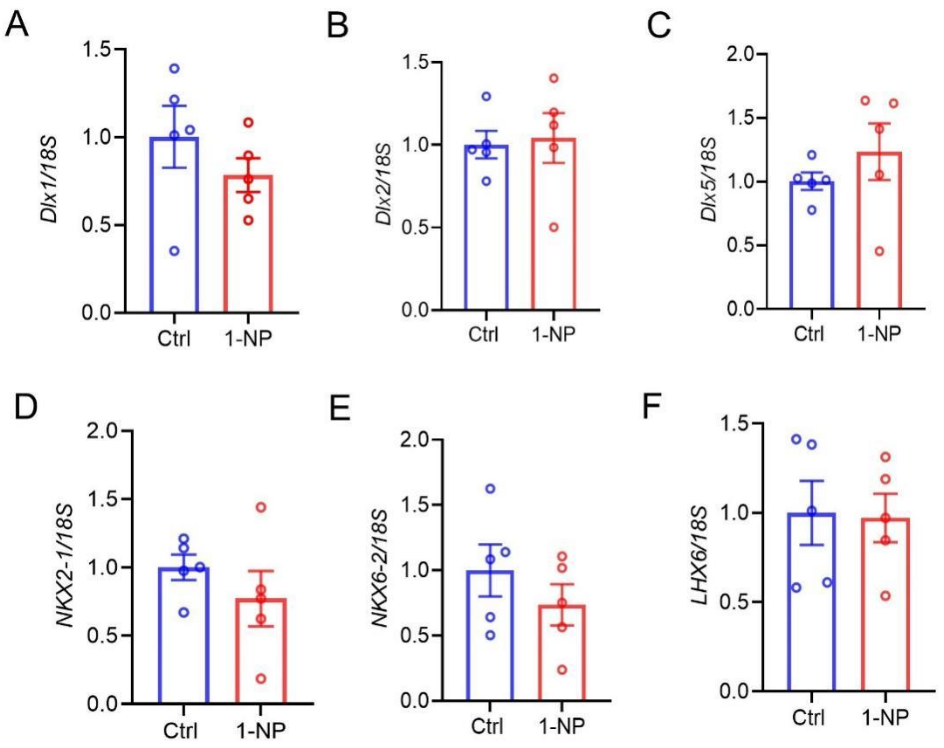

Fig.S2. Influence of gestational 1-NP exposure on the expression of differentiation related genes in forebrain interneuron. Differentiation-related genes in forebrain interneuron were detected in RT-PCR on GD14. N=5. (A) Dlx1. *t*=1.087, df=8, *P*=0.308. (B) Dlx2. *t*=0.232, df=8, *P*=0.822. (C) Dlx5. *t*=0.999, df=8, *P*=0.347. (D) NKX2-1. *t*=1.027, df=8, *P*=0.334. (E) NKX6-2. *t*=1.037, df=8, *P*=0.330. (F) LHX6. *t*=0.126, df=8, *P*=0.903.

Statistical analysis in the figure legends

Fig. 2. Influence of maternal 1-NP exposure on autism-like behavior in offspring.

	Groups		*t* value	df	*P* value
**Figure 2B**	1-NP (0 μg/kg)	S1 *vs* E	2.135	16	0.049
	1-NP (10 μg/kg)	S1 *vs* E	0.286	20	0.778
	1-NP (100 μg/kg)	S1 *vs* E	2.790	8.947	0.021
**Figure 2C**	1-NP (0 μg/kg)	S1 *vs* E	4.591	16	<0.01
	1-NP (10 μg/kg)	S1 *vs* E	2.395	13.52	0.032
	1-NP (100 μg/kg)	S1 *vs* E	0.416	20	0.682
**Figure 2E**	1-NP (0 μg/kg)	S1 *vs* S2	-2.296	16	0.036
	1-NP (10 μg/kg)	S1 *vs* S2	-2.017	15.057	0.052
	1-NP (100 μg/kg)	S1 *vs* S2	0.100	16	0.921
**Figure 2F**	1-NP (0 μg/kg)	S1 *vs* S2	-4.173	20	<0.01
	1-NP (10 μg/kg)	S1 *vs* S2	2.395	13.52	0.032
	1-NP (100 μg/kg)	S1 *vs* S2	-0.877	20	0.391
**Figure 2H**	1-NP (0 μg/kg)	S1 *vs* E	1.979	16	0.065
	1-NP (10 μg/kg)	S1 *vs* E	-0.045	20	0.965
	1-NP (100 μg/kg)	S1 *vs* E	-1.569	20	0.132
**Figure 2I**	1-NP (0 μg/kg)	S1 *vs* E	2.481	16	0.025
	1-NP (10 μg/kg)	S1 *vs* E	0.921	18	0.369
	1-NP (100 μg/kg)	S1 *vs* E	3.872	20	<0.01
**Figure 2K**	1-NP (0 μg/kg)	S1 *vs* S2	-2.275	16	0.037
	1-NP (10 μg/kg)	S1 *vs* S2	-0.676	20	0.507
	1-NP (100 μg/kg)	S1 *vs* S2	-0.358	20	0.724
**Figure 2L**	1-NP (0 μg/kg)	S1 *vs* S2	-4.221	16	<0.01
	1-NP (10 μg/kg)	S1 *vs* S2	-3.280	18	<0.01
	1-NP (100 μg/kg)	S1 *vs* S2	0.003	20	0.998

Fig. 3. Influence of maternal 1-NP exposure on mIPSC in offspring.

	Groups	*t* value	df	*P* value
**Figure 3B**	1-NP (0 μg/kg) *vs* 1-NP (100 μg/kg)	4.181	22	<0.01
**Figure 3C**	1-NP (0 μg/kg) *vs* 1-NP (100 μg/kg)	1.069	22	0.297
**Figure 3E**	1-NP (0 μg/kg) *vs* 1-NP (100 μg/kg)	0.145	22	0.887
**Figure 3F**	1-NP (0 μg/kg) *vs* 1-NP (100 μg/kg)	2.481	22	0.0212
**Figure 3H**	1-NP (0 μg/kg) *vs* 1-NP (100 μg/kg)	8.575	22	<0.01
**Figure 3I**	1-NP (0 μg/kg) *vs* 1-NP (100 μg/kg)	2.514	22	0.02
**Figure 3K**	1-NP (0 μg/kg) *vs* 1-NP (100 μg/kg)	0.815	22	0.424
**Figure 3L**	1-NP (0 μg/kg) *vs* 1-NP (100 μg/kg)	4.118	22	<0.01

Fig. 4. Influence of maternal 1-NP exposure on GAD67+ interneurons in weaning offspring.

	Groups	*t* value	df	*P* value
**Figure 4B**	1-NP (0 μg/kg) *vs* 1-NP (100 μg/kg)	2.558	6	0.043

**Figure 4D**		1-NP (0 µg/kg) *vs* 1-NP (100 µg/kg)	0.075	6	0.943
**Figure 4F**	Cg1	1-NP (0 µg/kg) *vs* 1-NP (100 µg/kg)	6.985	4.517	<0.01
	PrL	1-NP (0 µg/kg) *vs* 1-NP (100 µg/kg)	4.796	6	<0.01
	IL	1-NP (0 µg/kg) *vs* 1-NP (100 µg/kg)	6.245	3.576	<0.01
**Figure 4G**	Cg1	1-NP (0 µg/kg) *vs* 1-NP (100 µg/kg)	-0.872	6	0.417
	PrL	1-NP (0 µg/kg) *vs* 1-NP (100 µg/kg)	-0.264	6	0.8
	IL	1-NP (0 µg/kg) *vs* 1-NP (100 µg/kg)	0.115	6	0.912
**Figure 4I**		1-NP (0 µg/kg) *vs* 1-NP (100 µg/kg)	2.558	6	0.043
**Figure 4K**		1-NP (0 µg/kg) *vs* 1-NP (100 µg/kg)	0.075	6	0.943
**Figure 4M**	Cg1	1-NP (0 µg/kg) *vs* 1-NP (100 µg/kg)	6.735	3.859	<0.01
	PrL	1-NP (0 µg/kg) *vs* 1-NP (100 µg/kg)	3.202	3.685	0.037
	IL	1-NP (0 µg/kg) *vs* 1-NP (100 µg/kg)	2.728	4	0.052
**Figure 4N**	Cg1	1-NP (0 µg/kg) *vs* 1-NP (100 µg/kg)	-1.500	6	0.184
	PrL	1-NP (0 µg/kg) *vs* 1-NP (100 µg/kg)	-2.477	6	0.048
	IL	1-NP (0 µg/kg) *vs* 1-NP (100 µg/kg)	-0.647	6	0.541

Fig. 5. Influence of gestational 1-NP exposure on migration of interneurons in fetal brain.

	Groups		***t* value**	**df**	***P* value**
**Figure 5B**		1-NP (0 µg/kg) *vs* 1-NP (100 µg/kg)	3.421	4	0.027
**Figure 5D**	SVZ/VZ	1-NP (0 µg/kg) *vs* 1-NP (100 µg/kg)	-3.426	6	0.014
	IZ	1-NP (0 µg/kg) *vs* 1-NP (100 µg/kg)	0.110	6	0.916
	CP	1-NP (0 µg/kg) *vs* 1-NP (100 µg/kg)	3.279	6	0.017
	MZ	1-NP (0 µg/kg) *vs* 1-NP (100 µg/kg)	3.110	6	0.021
**Figure 5F**		1-NP (0 µg/kg) *vs* 1-NP (100 µg/kg)	2.820	4	0.048
**Figure 5H**	SVZ/VZ	1-NP (0 µg/kg) *vs* 1-NP (100 µg/kg)	-2.318	6	0.06
	IZ	1-NP (0 µg/kg) *vs* 1-NP (100 µg/kg)	0.460	6	0.662

	CP	1-NP (0 μg/kg) *vs* 1-NP (100 μg/kg)	6.401	6	<0.01
	MZ	1-NP (0 μg/kg) *vs* 1-NP (100 μg/kg)	4.267	6	<0.01
**Figure 5J**	1-NP (0 μg/kg) *vs* 1-NP (100 μg/kg)		0.287	6	0.784
**Figure 5L**	Bin 1	1-NP (0 μg/kg) *vs* 1-NP (100 μg/kg)	1.082	6	0.321
	Bin 2	1-NP (0 μg/kg) *vs* 1-NP (100 μg/kg)	2.392	6	0.054
	Bin 3	1-NP (0 μg/kg) *vs* 1-NP (100 μg/kg)	2.591	6	0.041
**Figure 5M**	Cxcl12	Ctrl *vs* 1-NP	0.456	8	0.660
**Figure 5N**	Cxcr4	Ctrl *vs* 1-NP	0.194	8	0.851
**Figure 5O**	Cxcr7	Ctrl *vs* 1-NP	2.090	8	0.07
**Figure 5P**	Slit1	Ctrl *vs* 1-NP	0.6086	8	0.547
**Figure 5Q**	Efna5	Ctrl *vs* 1-NP	0.036	8	.9718
**Figure 5R**	Arx	Ctrl *vs* 1-NP	0.507	8	0.625
**Figure 5S**	Nrp1	Ctrl *vs* 1-NP	1.279	8	0.237
**Figure 5T**	Nrp2	Ctrl *vs* 1-NP	0.291	8	0.778
**Figure 5U**	Sema3A	Ctrl *vs* 1-NP	0.765	8	0.466
**Figure 5V**	Nrg1	Ctrl *vs* 1-NP	2.477	8	0.038
**Figure 5W**	Erbb4	Ctrl *vs* 1-NP	3.353	8	0.01
**Figure 5X**	Sema3F	Ctrl *vs* 1-NP	2.494	8	0.0373

Fig. 6. Influence of gestational 1-NP exposure on hydroxymethylation of interneuron migration-related genes in fetal forebrain.

	Groups		***t* value**	**df**	***P* value**
**Figure 6B**	Nrg1_1	1-NP (0 μg/kg) *vs* 1-NP (100 μg/kg)	-0.268	8	0.795
	Nrg1_2	1-NP (0 μg/kg) *vs* 1-NP (100 μg/kg)	-0.679	8	0.516
	Erbb4	1-NP (0 μg/kg) *vs* 1-NP (100 μg/kg)	-0.358	8	0.73
	Sema3F_1	1-NP (0 μg/kg) *vs* 1-NP (100 μg/kg)	3.264	8	0.011
	Sema3F_2	1-NP (0 μg/kg) *vs* 1-NP (100 μg/kg)	-0.841	8	0.425
**Figure 6C**	Nrg1_2 (9246 site)	1-NP (0 μg/kg) *vs* 1-NP (100 μg/kg)	2.766	8	0.024
	Erbb4 (7743 site)	1-NP (0 μg/kg) *vs* 1-NP (100 μg/kg)	2.177	8	0.061
	Erbb4 (7866 site)	1-NP (0 μg/kg) *vs* 1-NP (100 μg/kg)	2.363	8	0.046

	Sema3F_1 (9269 site)	1-NP (0 μg/kg) *vs* 1-NP (100 μg/kg)	2.567	8	0.033
	Sema3F_1 (9348 site)	1-NP (0 μg/kg) *vs* 1-NP (100 μg/kg)	4.555	8	<0.01
	Sema3F_2 (0238 site)	1-NP (0 μg/kg) *vs* 1-NP (100 μg/kg)	2.577	8	0.033
**Figure 6D**	Tet1	1-NP (0 μg/kg) *vs* 1-NP (100 μg/kg)	-2.634	4.330	0.053
	Tet2	1-NP (0 μg/kg) *vs* 1-NP (100 μg/kg)	0.86	8	0.415
	Tet3	1-NP (0 μg/kg) *vs* 1-NP (100 μg/kg)	-2.250	8	0.055
**Figure 6F**	Tet1	1-NP (0 μg/kg) *vs* 1-NP (100 μg/kg)	1.448	6	0.1978
	Tet2	1-NP (0 μg/kg) *vs* 1-NP (100 μg/kg)	0.791	6	0.459
	Tet3	1-NP (0 μg/kg) *vs* 1-NP (100 μg/kg)	0.245	6	0.814
**Figure 6G**	1-NP (0 μg/kg) *vs* 1-NP (100 μg/kg)		3.400	8	<0.01

Fig. 7. Influence of gestational 1-NP exposure on mitochondrial function in fetal forebrain.

	**Groups**	***t* value**	**df**	***P* value**
**Figure 7H**	Ctrl *vs* 1-NP	0.263	6	0.802
**Figure 7I**	Ctrl *vs* 1-NP	2.890	6	0.027
**Figure 7K**	Ctrl *vs* 1-NP	4.133	6	<0.01
**Figure 7L**	Ctrl *vs* 1-NP	2.733	6	0.034
**Figure 7N**	Ctrl *vs* 1-NP	4.175	6	<0.01
**Figure 7O**	Ctrl *vs* 1-NP	2.791	6	0.024

Fig. 8. Effects of supplementation with α-KG on 1-NP-evoked hypohydroxymethylation of interneuron migration-related genes and interneuron migration inhibition in fetal forebrain.

	**Groups**	**F value**	**df**	***P* value**
**Figure 8A**	α-KG main effect	0.587	1	0.455
	1-NP main effect	20.039	1	<0.01
	α-KG*1-NP interaction	4.355	1	0.053
	Ctrl *vs* 1-NP	-	-	<0.01
	1-NP *vs* 1-NP+α-kG	-	-	0.061
**Figure 8B**	α-KG main effect	0.075	1	0.787
	1-NP main effect	1.671	1	0.214
	α-KG*1-NP interaction	1.759	1	0.203
	Ctrl *vs* 1-NP	-	-	0.083
	1-NP *vs* 1-NP+α-kG	-	-	0.274

**Figure 8C**		α-KG main effect	0.063	1	0.804
		1-NP main effect	12.403	1	<0.01
		α-KG*1-NP interaction	1.593	1	0.225
		Ctrl *vs* 1-NP	-	-	<0.01
		1-NP *vs* 1-NP+α-kG	-	-	0.300
**Figure 8D**		α-KG main effect	0.588	1	0.454
		1-NP main effect	5.353	1	0.034
		α-KG*1-NP interaction	9.544	1	<0.01
		Ctrl *vs* 1-NP	-	-	<0.01
		1-NP *vs* 1-NP+α-kG	-	-	0.120
**Figure 8E**		α-KG main effect	0.014	1	0.909
		1-NP main effect	1.785	1	0.200
		α-KG*1-NP interaction	1.512	1	0.237
		Ctrl *vs* 1-NP	-	-	0.088
		1-NP *vs* 1-NP+α-kG	-	-	0.443
**Figure 8F**		α-KG main effect	5.145	1	0.038
		1-NP main effect	3.712	1	0.072
		α-KG*1-NP interaction	10.504	1	<0.01
		Ctrl *vs* 1-NP	-	-	<0.01
		1-NP *vs* 1-NP+α-kG	-	-	<0.01
**Figure 8G**		α-KG main effect	0.199	1	0.662
		1-NP main effect	3.036	1	0.101
		α-KG*1-NP interaction	6.155	1	0.025
		Ctrl *vs* 1-NP	-	-	<0.01
		1-NP *vs* 1-NP+α-kG	-	-	<0.01
**Figure 8H**		α-KG main effect	0.040	1	0.843
		1-NP main effect	14.699	1	<0.01
		α-KG*1-NP interaction	5.187	1	0.037
		Ctrl *vs* 1-NP	-	-	<0.01
		1-NP *vs* 1-NP+α-kG	-	-	0.090
**Figure 8I**		α-KG main effect	0.634	1	0.438
		1-NP main effect	7.255	1	0.016
		α-KG*1-NP interaction	1.981	1	0.178
		Ctrl *vs* 1-NP	-	-	0.010
		1-NP *vs* 1-NP+α-kG	-	-	0.069
**Figure 8K**	Bin 1	α-KG main effect	1.473	1	0.248
		1-NP main effect	0.829	1	0.381
		α-KG*1-NP interaction	0.655	1	0.434
		Ctrl *vs* 1-NP	-	-	0.247
		1-NP *vs* 1-NP+α-kG	-	-	0.178
	Bin 2	α-KG main effect	13.963	1	<0.01
		1-NP main effect	2.509	1	0.139
		α-KG*1-NP interaction	2.258	1	0.159

	Ctrl *vs* 1-NP	-	-	0.050
	1-NP *vs* 1-NP+α-kG	-	-	<0.01
Bin 3	α-KG main effect	25.004	1	<0.01
	1-NP main effect	25.814	1	<0.01
	α-KG*1-NP interaction	2.033	1	0.179
	Ctrl *vs* 1-NP	-	-	<0.01
	1-NP *vs* 1-NP+α-kG	-	-	<0.01

Fig. 9. Effects of gestational α-KG supplementation on interneurons in weaning offspring.

		Groups	F value	df	*P* value
**Figure 9B**		α-KG main effect	3.062	1	0.118
		1-NP main effect	15.657	1	<0.01
		α-KG*1-NP interaction	5.158	1	0.052
		Ctrl *vs* 1-NP	-	-	<0.01
		1-NP *vs* 1-NP+α-kG	-	-	0.022
**Figure 9D**	Cg1	α-KG main effect	13.905	1	<0.01
		1-NP main effect	20.137	1	<0.01
		α-KG*1-NP interaction	12.084	1	<0.01
		Ctrl *vs* 1-NP	-	-	<0.01
		1-NP *vs* 1-NP+α-kG	-	-	<0.01
	PrL	α-KG main effect	4.842	1	0.048
		1-NP main effect	16.70	1	<0.01
		α-KG*1-NP interaction	3.798	1	0.075
		Ctrl *vs* 1-NP	-	-	<0.01
		1-NP *vs* 1-NP+α-kG	-	-	0.013
	IL	α-KG main effect	2.803	1	0.120
		1-NP main effect	11.212	1	<0.01
		α-KG*1-NP interaction	2.803	1	0.120
		Ctrl *vs* 1-NP	-	-	<0.01
		1-NP *vs* 1-NP+α-kG	-	-	0.036
**Figure 9E**	Cg1	α-KG main effect	0.004	1	0.953
		1-NP main effect	0.613	1	0.449
		α-KG*1-NP interaction	0.033	1	0.860
		Ctrl *vs* 1-NP	-	-	0.509
		1-NP *vs* 1-NP+α-kG	-	-	0.934
	PrL	α-KG main effect	1.738	1	0.212
		1-NP main effect	0.679	1	0.426
		α-KG*1-NP interaction	0.033	1	0.860
		Ctrl *vs* 1-NP	-	-	0.431
		1-NP *vs* 1-NP+α-kG	-	-	0.498

	IL	α-KG main effect	0.123	1	0.732
		1-NP main effect	0.123	1	0.732
		α-KG*1-NP interaction	0.204	1	0.660
		Ctrl *vs* 1-NP	-	-	0.945
		1-NP *vs* 1-NP+α-kG	-	-	0.581
**Figure 9G**		α-KG main effect	1.171	1	0.311
		1-NP main effect	6.809	1	0.031
		α-KG*1-NP interaction	3.973	1	0.081
		Ctrl *vs* 1-NP	-	-	0.012
		1-NP *vs* 1-NP+α-kG	-	-	0.061
**Figure 9I**	Cg1	α-KG main effect	4.757	1	0.050
		1-NP main effect	42.971	1	<0.01
		α-KG*1-NP interaction	1.255	1	0.285
		Ctrl *vs* 1-NP	-	-	<0.01
		1-NP *vs* 1-NP+α-kG	-	-	0.038
	PrL	α-KG main effect	0.062	1	0.807
		1-NP main effect	17.266	1	<0.01
		α-KG*1-NP interaction	1.813	1	0.203
		Ctrl *vs* 1-NP	-	-	<0.01
		1-NP *vs* 1-NP+α-kG	-	-	0.281
	IL	α-KG main effect	3.286	1	0.095
		1-NP main effect	17.217	1	<0.01
		α-KG*1-NP interaction	2.199	1	0.164
		Ctrl *vs* 1-NP	-	-	<0.01
		1-NP *vs* 1-NP+α-kG	-	-	0.038
**Figure 9J**	Cg1	α-KG main effect	0.778	1	0.395
		1-NP main effect	1.130	1	0.309
		α-KG*1-NP interaction	0.001	1	0.982
		Ctrl *vs* 1-NP	-	-	0.476
		1-NP *vs* 1-NP+α-kG	-	-	0.534
	PrL	α-KG main effect	0.046	1	0.834
		1-NP main effect	7.292	1	0.019
		α-KG*1-NP interaction	1.546	1	0.237
		Ctrl *vs* 1-NP	-	-	0.481
		1-NP *vs* 1-NP+α-kG	-	-	0.323
	IL	α-KG main effect	0.601	1	0.453
		1-NP main effect	0.082	1	0.779
		α-KG*1-NP interaction	0.082	1	0.779
		Ctrl *vs* 1-NP	-	-	0.692
		1-NP *vs* 1-NP+α-kG	-	-	0.467

Fig. 10. Effects of gestational α-KG supplementation on 1-NP-induced mIPSC transmission disorder and autism-like behaviors.

	Groups		F value	df	*P* value
**Figure 10B**	α-KG main effect		5.135	1	0.028
	1-NP main effect		35.559	1	<0.01
	α-KG*1-NP interaction		3.084	1	0.085
	Ctrl *vs* 1-NP		-	-	<0.01
	1-NP *vs* 1-NP+α-kG		-	-	<0.01
**Figure 10C**	α-KG main effect		1.611	1	0.210
	1-NP main effect		8.726	1	<0.01
	α-KG*1-NP interaction		0.012	1	0.913
	Ctrl *vs* 1-NP		-	-	0.050
	1-NP *vs* 1-NP+α-kG		-	-	0.335
**Figure 10E**	α-KG main effect		0.140	1	0.710
	1-NP main effect		0.002	1	0.968
	α-KG*1-NP interaction		0.002	1	0.962
	Ctrl *vs* 1-NP		-	-	0.951
	1-NP *vs* 1-NP+α-kG		-	-	0.767
**Figure 10F**	α-KG main effect		0.033	1	0.858
	1-NP main effect		9.319	1	<0.01
	α-KG*1-NP interaction		6.794	1	0.012
	Ctrl *vs* 1-NP		-	-	<0.01
	1-NP *vs* 1-NP+α-kG		-	-	0.093
**Figure 10G**	Ctrl	S1 *vs* E	2.893	18	0.01
	α-kG	S1 *vs* E	0.147	20	0.044
	1-NP	S1 *vs* E	1.566	18	0.135
	1-NP+α-kG	S1 *vs* E	2.356	16	0.032
**Figure 10H**	Ctrl	S1 *vs* S2	-3.900	18	<0.01
	α-kG	S1 *vs* S2	-3.787	20	<0.01
	1-NP	S1 *vs* S2	0.833	18	0.416
	1-NP+α-kG	S1 *vs* S2	-6.918	16	<0.01
**Figure 10I**	Ctrl	S1 *vs* E	3.858	18	<0.01
	α-kG	S1 *vs* E	2.371	18	0.029
	1-NP	S1 *vs* E	1.292	16	0.215
	1-NP+α-kG	S1 *vs* E	2.704	18	0.015
**Figure 10J**	Ctrl	S1 *vs* S2	-3.782	13.042	<0.01
	α-kG	S1 *vs* S2	-2.256	11.934	0.044
	1-NP	S1 *vs* S2	-1.280	16	0.219
	1-NP+α-kG	S1 *vs* S2	-3.705	18	<0.01

Table S1 Primers for Multiplex PCR in measurement of 5hmC

Genes	Sequences
Nrg1_1	Forward: AGATTTAGAGGATTYGGGAGGA<br>Reverse: ACCCRAACCCAAATAAATACCA
Nrg1_2	Forward: GYGATAAGTTTGGTTTAAGGGTTTGTAG<br>Reverse: CCCRAAACACCCCCAAAC
Erbb4	Forward: GGGTTTAYGGGTTTTGGAAGT<br>Reverse: ACTCCCCCAAACCCAAAA
Sema3F_1	Forward: TTGYGGGGGTGGAAAAAG<br>Reverse: CCTCCCRACCCCACTAAAA
Sema3F_2	Forward: GGGTAGGGTTTAGGGTTTTTAGG<br>Reverse: CCAACCCTTCTCACCAAAAA

Table S2 Primers for real-time RT-PCR

Genes	Sequences
18S	Forward: GTAACCCGTTGAACCCCATT<br>Reverse: CCATCCAATCGGTAGTAGCG
Tet1	Forward: ACACAGTGGTGCTAATGCAG<br>Reverse: AGCATGAACGGGAGAATCGG
Tet2	Forward: AGAGAAGACAATCGAGAAGTCGG<br>Reverse: CCTTCCGTACTCCCAAACTCAT
Tet3	Forward: GTAACCCGTTGAACCCCATT<br>Reverse: CCATCCAATCGGTAGTAGCG
Erbb4	Forward: GGAACAGCAGTACCGAGCCTTG<br>Reverse: GGATAGACCGCAGGAAGGAGAGG
Nrg1	Forward: GCTCATCACTCCACGACTGTCAC<br>Reverse: CTGCTGTGCCTGCTGTTCTCTAC
Cxcl12	Forward: ACCAGTCAGCCTGAGCTACCG<br>Reverse: AAGGGCACAGTTTGGAGTGTTGAG
Cxcr4	Forward: GTCAACCTCTACAGCAGCGTTCTC<br>Reverse: CTGACTGTTGGTGGCGTGGAC
Sema3A	Forward: TGGGACGGGACTTCGCTATCTTC<br>Reverse: GGGATGAGATGGGCACTGATGAATC
Sema3F	Forward: GACCTGCATGACATCAACCGAGAG<br>Reverse: CTCCATTGCCATCCTTGCCTGAC
Nrp1	Forward: ACAGCATCCAATCAAGCCGACAG<br>Reverse: TCTTCTCATCTCCCAGGTCCACTTG
Nrp2	Forward: ATCAGTGCCTCCTCCACCTTCTC<br>Reverse: TGTCCAGCCATTGTCATCACCATG
Slit1	Forward: GCTACGCCTGCCTCTGTGTTG<br>Reverse: GGTCTACGCAGTTGGCTCCATTC
Efna5	Forward: ATGAGTCAGCCGAGCCATCCC

	Reverse: AGCATCGCCAGGAGGAACAGTAG
Arx	Forward: AGGGCAAGGATGGTGAGGACAG
	Reverse: GCTGGTAACTGGTGAACGTGGTG
Dlx1	Forward: ACCACCATGCCAGAAAGTCTCAAC
	Reverse: GCCCGCCGAGTGTAAACAGTG
Dlx2	Forward: CAACGAGCCGGACAAGGAAGAC
	Reverse: CTGGAGTAGATGGTGCGTGGTTTC
Dlx5	Forward: GCGACTTCCAAGCTCCGTTCC
	Reverse: AAGCAGAGGTAGGAGAGCAGTAGC
NKX2-1	Forward: CTCAGCCGACGCCGAATCATG
	Reverse: GCCCTCCATGCCCACTTTCTTG
NKX6-2	Forward: AAGTGAAGGTGTGGTTCCAGAATCG
	Reverse: CCGGTTGTATTCGTCATCGTCCTC
LHX6	Forward: TCTGGACAAGGACGAAGGTAGAGC
	Reverse: CAGACCGCAACTGGAGCAGATATTC
